# Long Term Followup of Photorefractive Keratectomy with Adjuvant Use of Mitomycin C

**DOI:** 10.1155/2014/821920

**Published:** 2014-04-29

**Authors:** Vasilios F. Diakonis, Vardhaman P. Kankariya, George D. Kymionis, Georgios Kounis, George Kontadakis, Eirineos Gkenos, Michael A. Grentzelos, George Hajithanasis, Sonia H. Yoo, Ioannis G. Pallikaris

**Affiliations:** ^1^Department of Ophthalmology, University Hospital of Heraklion, Heraklion, 71100 Crete, Greece; ^2^Bascom Palmer Eye Institute, Miller School of Medicine, University of Miami, Miami, FL 33136, USA

## Abstract

*Purpose.* To study the long term refractive and visual outcomes of photorefractive keratectomy (PRK) with intraoperative application of mitomycin C (MMC). * Methods.* This study included 37 eyes who received myopic PRK; after photoablation, a sponge soaked in 0.02% MMC solution was applied in all corneas for 2 minutes. Efficacy, safety, predictability, and stability of PRK MMC were evaluated. Endothelial cell density was evaluated at the last postoperative interval. * Results.* Mean preoperative spherical equivalent (SEQ) was −6.03 ± 1.87 D (diopters) and reduced to −0.09 ± 0.53 D at the last postoperative examination. Mean followup was 44.73 ± 18.24 months. All the eyes were in the ±1.00 D of attempted versus achieved SEQ at the one-year follow-up interval. Furthermore, 95% of the eyes did not lose lines or gained 1 to 2 lines of CDVA, while 5% lost 1 line. At the third postoperative month, 89% of the eyes either were clear or had trace haze, while 4 eyes had mild haze; by the 12-month postoperative interval, none of the eyes demonstrated haze. Mean endothelial cell density (ECD) at the last postoperative interval was 2658 ± 153 cells/mm^2^. * Conclusions.* PRK, with intraoperative use of MMC, demonstrates stable refractive and visual outcomes up to 44 months after surgery.

## 1. Introduction

Although LASIK is the most popular corneal refractive procedure performed today, PRK remains an excellent option for low to moderate myopia and low to moderate astigmatism [1]. In some cases, PRK may be preferable to LASIK, such as in patients with inadequate corneal thickness (concerns for postoperative corneal ectasia) or preexistent corneal surface pathology. Furthermore, some patients may even prefer PRK due to the possibility of flap related complications.

PRK has an excellent safety profile; the main drawback of surface corneal ablations for intermediate and high myopia is the higher possibility for keratocyte activation which may lead to visually significant corneal opacification (haze) and regression of the refractive outcomes [2–4]. During the last decade, several attempts have been made to improve PRK outcomes avoiding haze formation and regression, the most clinically effective being intraoperative use of mitomycin C (MMC) [5, 6].

Nine years after experimental studies on rabbit corneas [7], the first clinical study of PRK with adjuvant MMC in 2000 demonstrated satisfactory refractive outcomes by modulating corneal healing and controlling haze formation [8]. Mitomycin belongs to a group of synthetic medicines that have been derived from compounds of certain bacteria and fungi medicines and are called cytotoxic antibiotics. Mitomycin acts as an alkylating agent that inhibits DNA and protein synthesis by inserting itself into the strands of genetic material. Consequently, proliferation of rapidly growing cells such as fibroblasts is inhibited causing cell apoptosis. Attributable to its inhibiting properties, MMC has been used in ophthalmology over twenty years as an adjunctive treatment of a variety of ophthalmic conditions. Improvements in the outcomes of trabeculectomy [9], pterygium surgery [10], and corneal intraepithelial neoplasia [11] after the application of MMC have been reported extensively.

The purpose of this study is to investigate retrospectively the long term visual and refractive outcomes along with complications of photorefractive keratectomy with intraoperative application of MMC.

## 2. Patients and Methods

### 2.1. Patient Population

This retrospective clinical study includes patients who received myopic PRK treatment, using the 200 Hz Allegretto laser platform (Wavelight Laser Technologie AG, Erlangen, Germany), between March 2003 and March 2005. Inclusion criteria were healthy myopic patients 18 years of age or older (myopia less than −10.00 D with astigmatism less than 2.00 D), attempted optical treatment zone 6.5 mm, and two-minute intraoperative MMC exposure.

Twenty-four patients (37 eyes) were included in this study (8 males and 16 females), aged 20 to 55 (mean age: 34.13 ± 7.6). Mean preoperative SEQ was −6.03 + 1.87 D (range: −9.75 to −2.75 D).

### 2.2. Clinical Examination

A complete ophthalmic examination was performed preoperatively in all patients including manifest refraction, cycloplegic manifest refraction, corneal topography, central corneal pachymetry (50 M-Hz; Corneo-GAGE; Sonogage Inc., Cleveland, Ohio, USA), and biomicroscopy. Patients with signs of ocular disease such as active anterior segment disease, previous intraocular or corneal surgery, history of herpes keratitis, diagnosed autoimmune disease, systemic connective tissue disease or atopic syndrome, and corneal topographic findings suspicious for keratoconus were excluded.

All patients were appropriately informed of risks and benefits prior to operation, and they gave a written informed consent in accordance with the institutional guidelines and the Declaration of Helsinki.

### 2.3. Surgical Technique

All PRK procedures followed the same surgical technique by the same experienced surgeon. Two minutes after topical corneal anesthesia, mechanical epithelial debridement of the central 7.5 mm of the cornea (previously marked with a 7.5 mm epithelial trephine) was accomplished using a rotating soft brush [12] followed by a myopic photoablation performed using the Wavelight Allegretto laser 200 Hz. After photoablation, a merocel sponge soaked in MMC 0.02% solution was applied to the corneal stroma for two minutes and irrigation using 30 mL of balanced salt solution followed.

At the end of the procedure, a combination steroid and antibiotic drop (Tobradex, 4 times daily) was administered in all patients and a bandage soft contact lens was kept in place until full corneal reepithelialization occurred. After reepithelialization, patients were treated with fluorometholone sodium 2% (FML, Allergan, 2 times daily for four weeks).

### 2.4. Follow-up Examinations

Preoperative and postoperative followup (1-, 3-, 6-, and 12-month and last postoperative intervals) included uncorrected distance visual acuity (UDVA), corrected distance visual acuity (CDVA), manifest refraction, corneal topography, and complications.

Anterior stromal haze for PRK patients was graded subjectively during slit lamp biomicroscopy and was reported as one of five standardized categories described by Fantes: clear (grade 0), trace (haze seen only with broad-beam illumination, grade 0.5), mild (haze visible by slit-beam illumination, grade 1), moderate (haze somewhat obscuring iris detail, grade 2), marked (haze markedly obscuring iris detail, grade 3), and severe (completely opaque stroma in the area of ablation, grade 4).

Endothelial cell density was evaluated at the last postoperative interval using specular microscopy (Tomey, Japan).

### 2.5. Statistical Analysis

For the analysis of the results, we used Microsoft Excel 2007 and SPSS 17. Analysis of variance and independent* t*-test were used for estimating differences between groups. A* P* value less than 0.05 was regarded statistically significant.

## 3. Results

Mean followup was 44.73 ± 18.24 months (range: 26 to 59 months). Mean preoperative SEQ refraction was –6.03 ± 1.87 D (range from −9.75 to –2.75 D). Mean preoperative corneal pachymetry was 515 ± 24 *μ*m (range from 481 to 586 *μ*m). Mean preoperative LogMAR CDVA was 0.00 ± 0.06 (range from 0.24 to −0.12) (20/20 Snellen).

### 3.1. Predictability

**Figure 1:**
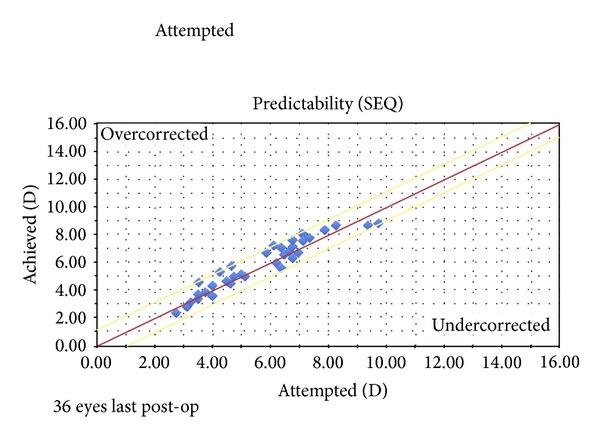
Predictability scattergram showing achieved versus attempted refractive correction at last postoperative examination.

**Figure 2:**
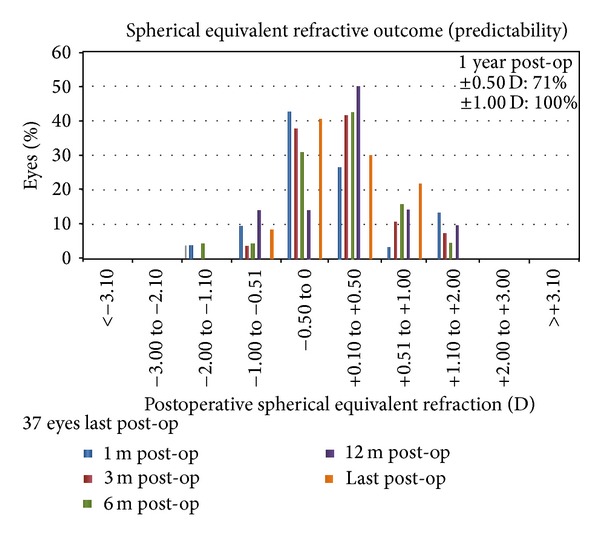
Spherical equivalent refractive outcome bar graph at all postoperative intervals (1, 3, 6, 12, and last postoperative examination).

The mean SEQ refraction reduced from –6.03 ± 1.87 D to 0.06 ± 0.72 D, 0.20 ± 0.45 D, 0.24 ± 0.60 D, −0.27 ± 0.70 D, and −0.09 ± 0.53 D at 1, 3, 6, 12, and last postoperative intervals, respectively. The standard deviation was below or equal to ±1.0 D for all periods except for the first postoperative month. All eyes were inside the 1.00 D region (Figure 1). Seventy percent of eyes were within the ±0.50 D and 100% within the ±1.00 D as shown in Figure 2.

### 3.2. Efficacy

**Figure 3:**
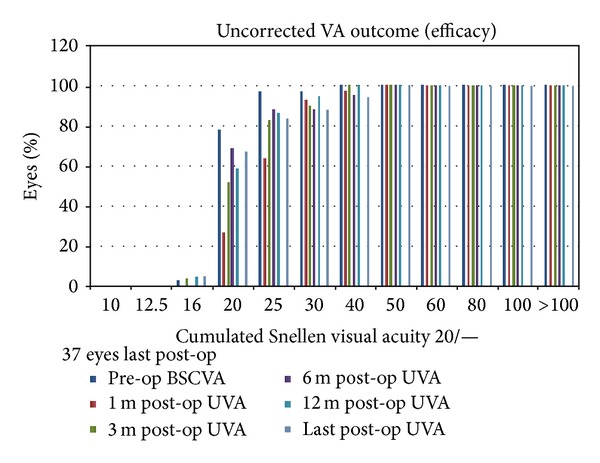
Uncorrected visual acuity bar graph at all postoperative intervals (1, 3, 6, 12, and last postoperative examination).

Preoperatively, all patients included in the study had 20/40 (0.3 LogMAR) or better CDVA. The post-op UDVA in Figure 3 is a cumulative graph of uncorrected visual acuities after surgery. At 1 month UVA was 20/25 (0.09 ± 0.09 LogMAR), at 3 months it was 20/22 (0.04 ± 0.08 LogMAR), at 6 months it was 20/22 (0.03 ± 0.11 LogMAR), at 12 months it was 20/21 (0.03 ± 0.09 LogMAR), and at the last postoperative interval it was 20/22 (0.04 ± 0.12 LogMAR).

### 3.3. Safety

**Figure 4:**
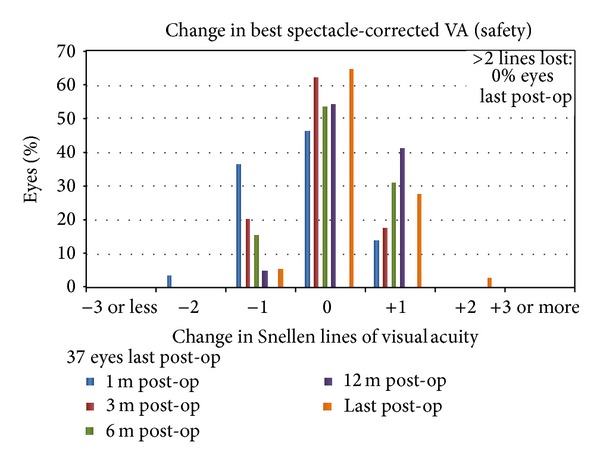
Change in spectacle corrected visual acuity bar graph at all postoperative intervals (1, 3, 6, 12, and last postoperative examination).

At one-month postoperative period one eye (3%) lost 2 lines of CDVA, while in the following postoperative periods this percentage reduced to 0 eyes (0%). At the last postoperative period 2 eyes (5%) lost 1 line, 24 eyes (65%) lost no lines, 10 eyes (27%) gained 1 line, and 1 eye (3%) gained 2 lines of CDVA.  Figure 4 is presenting the change in spectacle corrected visual acuity.

### 3.4. Stability

**Figure 5:**
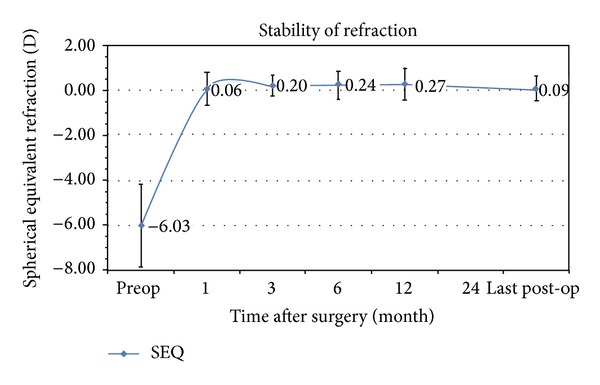
Stability of refraction bar (mean spherical equivalent). Error bars indicate standard deviation at each postoperative interval (1, 3, 6, 12, and last postoperative examination).

The mean preoperative SEQ refraction of –6.03 D decreased to 0.06 D at 1 month. At 3 months it was 0.20 D, at 6 months it was 0.24 D, at 12 months it was 0.27 D, and at last postoperative follow-up examination it was 0.09 D. The percentage of eyes with difference between 3 months and 12 months above 0.50 D was 6% and remained 6% for the period between 6 months and 12 months (Figure 5).

### 3.5. Adverse Effects and Postoperative Complications

No intra- or postoperative complications, such as delayed epithelial healing, or infections were found. Epithelial healing ranged from 3 to 5 days in all eyes (mean time 3.9 ± 0.7 days).

At the third post-PRK MMC month, 33 eyes (89%) either were clear (26 eyes, 70%) or had trace haze (7 eyes, 19%), while 4 eyes (11%) had mild haze (no eye had moderate or marked haze). Progressive corneal clearing occurred over subsequent months. At the sixth post-PRK month, only 4 eyes (11%) had trace haze. At the last postoperative interval, all corneas were found clear.

Mean endothelial cell density (ECD) at the last postoperative interval was 2658 ± 153 cells/mm^2^, while none of the eyes demonstrated an ECD value of less than 2000.

## 4. Discussion

Mitomycin C is used commonly today after photorefractive keratectomy to modulate the corneal wound healing response and prevent occurrence of primary as well as recurrence of preexisting haze formation [5, 8]. The original protocol of intraoperative MMC exposure suggested a two-minute time interval [8]; the same protocol was also used in all patients of the current study. Due to concerns regarding MMCs safety [13], a series of alterations on the usage of MMC have been suggested [14]. This has led to exposure time reduction of MMC with the aim of achieving an equivalent effect on haze inhibition but with less potential toxic effects. Current studies demonstrate haze inhibition with an intraoperative MMC exposure interval which varies from 15 to 120 seconds (depending on the attempted correction) [15–17]. Nevertheless, a nomogram of MMC exposure time and attempted correction has yet been described.

In our study, we evaluated retrospectively the refractive and visual outcomes of the first patients treated for myopia using PRK with adjuvant MMC (two-minute exposure) in our refractive surgery center. The results demonstrate stability over a 44-month period, while no intraoperative or early or late postoperative complications were found. Furthermore, the procedure seems safe, since at the last postoperative interval 95% of eyes either did not lose or gained 1 to 2 lines of CDVA, and only 5% lost 1 line of CDVA.

Corneal haze inhibition after PRK was accomplished in this group of patients. MMC was used immediately after photoablation for two minutes; the exposure time of MMC has been decreased over the past years (haze inhibition has been demonstrated with a 15-second MMC exposure) [15] due to concerns about possible toxic effects that it may imply different ocular tissues [18, 19]. These toxic effects are directly associated with the drug penetration and deposition when it is used in extraocular procedures such as episcleral application [20], while MMCs penetration level is associated with its concentration in the solution used and the time duration applied on the eye [21].

A two-minute MMC exposure time during PRK is today only used for specific cases such as retreatments in corneas with postoperative haze and treatments of buttonhole complications during LASIK (laser* in situ* keratosmileusis) [22], while the common intraoperative exposure of MMC does not exceed 30 seconds during PRK. This study reveals that the original protocol of MMC use (two minutes) does not demonstrate any early or late (up to 44 months postoperatively) complications and thereby it seems that it is safe for other intraoperative applications.

Endothelial cell density changes after PRK MMC have been controversial in previous studies [15, 23, 24]. Even though we did not compare ECD with preoperative values as they were not obtained (toxicity issues were raised later in time in respect to the operation dates and ECD assessment was not a standard examination), we found mean ECD at the last postoperative interval to be within the normal limits. Furthermore, all patients had an ECD no less than 2000 cells/mm^2^. This finding alone may suggest that no evident MMC induced toxic effect was revealed in this patient group up to 44 months after surgery; nevertheless, no conclusive statements with respect to possible induced toxic effects may be presented by the current study.

There are several limitations in this study. First, the small number of eyes studied; furthermore, the study was uncontrolled. A control group of eyes that have undergone the PRK using the same surgical technique without adjuvant MMC would have provided a means to compare the long term outcomes between PRK and PRK MMC procedures. Nevertheless, PRK outcomes presented in previous studies are comparable to our results [25]. Another limitation of the current study is that we did not examine possible changes in corneal endothelial cell density (ECD) of these patients (comparison between preoperative ECD and postoperative ECD) in order to exclude any possible MMC related toxic effects at the level on the endothelial cells. Nevertheless, we have an ECD at the last postoperative interval revealing normal values.

In conclusion, PRK with intraoperative 0.02% MMC for 2 minutes had good predictability and safety for up to 44-month followup without progressive time dependent sight threatening complications.
